# Prolonged Active Prone Positioning in Spontaneously Breathing Non-intubated Patients With COVID-19-Associated Hypoxemic Acute Respiratory Failure With PaO_2_/FiO_2_ >150

**DOI:** 10.3389/fmed.2021.626321

**Published:** 2021-07-21

**Authors:** Paola Pierucci, Nicolino Ambrosino, Valentina Di Lecce, Michela Dimitri, Stefano Battaglia, Esterina Boniello, Andrea Portacci, Onofrio Resta, Giovanna Elisiana Carpagnano

**Affiliations:** ^1^A. Cardiothoracic Department, Respiratory and Critical Care Unit Bari Policlinic University Hospital, B. Section of Respiratory Diseases, Department of Basic Medical Science Neuroscience and Sense Organs, University of Bari ‘Aldo Moro’, Bari, Italy, University of Bari, Bari, Italy; ^2^Istituti Clinici Scientifici Maugeri Istituto di Ricovero e Cura a Carattere Scientifico Pneumologia Riabilitativa, Istituto di Montescano, Montescano, Italy; ^3^Department of Emergency and Organ Transplantation, University of Bari, Bari, Italy

**Keywords:** COVID-19, prone position, non-intubated, spontaneously breathing, hypoxic respiratory failure

## Abstract

**Background:** The COVID-19 pandemic has led to new approaches to manage patients outside the ICU, including prone positioning in non-intubated patients.

**Objectives:** To report the use of prolonged active prone positioning in spontaneously breathing patients with COVID-19-associated acute respiratory failure. Spontaneously breathing vs non-invasive respiratory support for COVID19 associated acute respiratory failure.

**Methods:** Patients with PaO_2_/FiO_2_ > 150, with lung posterior consolidations as assessed by means of lung ultrasound, and chest x-ray were studied. Under continuous pulse oximetry (SpO_2_) monitoring, patients maintained active prone position. A PaO_2_/FiO_2_ < 150 was considered as treatment failure and patients had to be switched to non-invasive respiratory support. Retrospectively, data of 16 patients undergoing who refused proning and underwent non-invasive respiratory support were used as controls. The primary outcome was the proportion of patients maintaining prolonged prone position and discharged home. Secondary outcomes included improvement in oxygenation, hospital length of stay, and 6-month survival.

**Results:** Three out of 16 (18.7%) patients did not tolerate the procedure. Three more patients showed a worsening in PaO_2_/FiO_2_ to <150 and required non-invasive support, two of whom finally needing endotracheal intubation. After 72 h, 10 out of 16 (62.5%) patients improved oxygenation [PaO_2_/FiO_2_: from 194.6 (42.1) to 304.7 (79.3.2) (*p* < 0.001)] and were discharged home. In the control group, three out of 16 failed, required invasive ventilatory support, and died within 1 month in ICU. Thirteen were successful and discharged home.

**Conclusion:** In non-intubated spontaneously breathing COVID-19 patients with PaO_2_/FiO_2_ >150, active prolonged prone positioning was feasible and tolerated with significant improvement in oxygenation.

## Introduction

The Coronavirus disease 19 (COVID-19) pandemic has led to more than 1 million casualties worldwide (1). COVID-19 severely hit Italy with an increasingly number of patients admitted to hospitals with hypoxemic acute respiratory failure (ARF) needing intensive care unit (ICU) admission, often exceeding the availability of ICU beds. Due to ICU bed and ventilator shortage, critical care resources were saved to care for patients with ARF also outside the ICU (2, 3).

Mechanical ventilation in the prone position (PP) is a validated strategy in the treatment of acute respiratory distress syndrome (ARDS) (4) with several beneficial effects on pulmonary physiology. In supine position, pulmonary edema accumulates in basal regions, leading to increased air volume delivered to apical and anterior lung units, which are also the regions receiving less pulmonary circulation. Prone positioning leads to more homogeneous distribution of ventilation, thus decreasing the shunt fraction and improving matching of ventilation and perfusion (4). Recently, studies have reported improvement in oxygenation during PP in non-intubated awake patients with COVID-19 during spontaneous and non-invasively assisted breathing (5–8, 8–11). However, those were mainly physiologic studies of active PP for a few daily hours. We aimed to report the feasibility and the tolerability of prolonged active PP in non-intubated, spontaneously breathing patients with arterial oxygen tension to inspiratory oxygen fraction ratio (PaO_2_/FiO_2_) > 150 in a respiratory intensive care unit (RICU). Secondary outcomes were PaO_2_/FiO_2_ before and after the trial and during PP, the length of stay (LoS) in hospital, and 1- and 6-month survival rates.

## Methods

This observational, prospective, single-center study was approved by the Bari Policlinico ethic committee (study number 6363), and written or verbal informed consent was obtained from all patients.

### Patients

From March 11, to April 30, 2020, all consecutive patients with confirmed COVID-19 without indication for immediate non-invasive respiratory support (NRS) were considered eligible if they had (1) PaO_2_/FiO_2_ > 150 and (2) lung ultrasound (LUS) and chest x-ray signs of basal and posterior consolidations. Under continuous pulse oximetry (SpO_2_) and EKG monitoring and oxygen supplementation to maintain SpO_2_ > 96%, spontaneously breathing patients were asked to actively maintain PP (Study Group). Active PP had to be maintained as longest as possible with intervals for meals and other personal care.

Patients who refused to undergo PP were initiated to NRS either with high-flow nasal cannula (HFNC), continuous positive airway pressure (CPAP), or non-invasive ventilation (NIV). These patients with the same clinical and physiological characteristics as the study group were retrospectively considered as controls.

One or more of the following drugs were administered: hydrosi-chloroquine, macrolides, steroids, anticoagulants, tocilizumab, N-acetyl-cysteine, vitamin D, and vitamin C (12), and the rest of patients' comorbidities treatment.

### Measurements

Demographics, clinical characteristics, comorbidities, the sequential organ failure assessment (SOFA), and 1- and 6-month survival were recorded. Chest x-ray and LUS were assessed at admission and at discharge. The CT chest was not promptly available in the same location; therefore, considering the severity of the hypoxic ARF, it was not performed in all patients.

The study group underwent arterial blood gas analysis at the 6th, 24th, 48th, and 72nd hour during PP and in supine position before and after the trial. Controls underwent arterial blood gas analysis at the start of the NRS and at discharge from the RICU.

In patients of the study group, the ventilatory ratio (VR), an index of respiratory effort, was calculated according to Morales-Quinteros et al. (13).

Worsening in PaO_2_/FiO_2_ to <150 was considered as treatment failure. Patients in the PP group who dropped out or who were failing treatment were switched to NRS. Control patients failing NRS were transferred to the ICU for invasive ventilator support.

### Procedure

**Figure 1:**
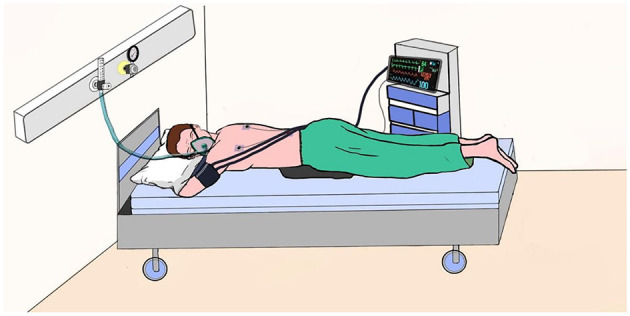
Cartoon demonstrating continuous prone positioning with oxygen supplementation only *via* Venturi Mask demonstrated under continuous monitoring.

The nurse-to-patient ratio in the RICU was 1:4 to 1: 5. Patients enrolled maintained PP with the Venturi Oxygen mask under non-invasive monitoring of EKG, SpO_2_, and arterial blood pressure. During PP, EKG patches were moved from the front to the back of the patients' chest. All healthcare providers wore personal protective equipment, whereas all patients were wearing a surgical mask on top of the Venturi mask (14, 15) (Figure 1).

Control patients continuously performed NRS in semi-recumbent supine position, with only short intervals for eating and personal care. Patients on HFNC were wearing a surgical mask on top of nasal cannula, whereas those on CPAP and NIV used masks closed without expiratory holes and an antiviral filter was inserted between the mask and the circuit to filter all patients' exhaled air.

### Statistical Analysis

Statistical analysis was performed using the R statistical environment (16). For each variable, Shapiro–Wilk test and graphical evaluations (Q–Q plots) were applied to assess the correspondence with the normal distribution. Descriptive statistics are shown as mean ± standard deviation (SD) and/or median and interquartile range [IQR] for normally distributed and non-normally distributed continuous variables, respectively, whereas categorical variables were indicated with absolute frequency (%). Student's *t*-test and Mann–Whitney *U*-test were performed to assess between group comparisons, as appropriate. In particular, to evaluate differences between PaO_2_/FiO_2_ at admission and discharge, a paired *t*-test was used. Differences for categorical variables between groups were assessed by a Pearson χ^2^ test and or Fisher's exact test. Then, multivariate ANOVA and ANCOVA analyses were performed to compare PaO_2_/FiO_2_ at discharge between the two experimental groups, namely, PaO_2_/FiO_2_ at admission as a covariate. *p*-values lower than 0.05 were regarded as significant. Plots and graphs were realized using the Excel graph tool.

## Results

**Figure 2:**
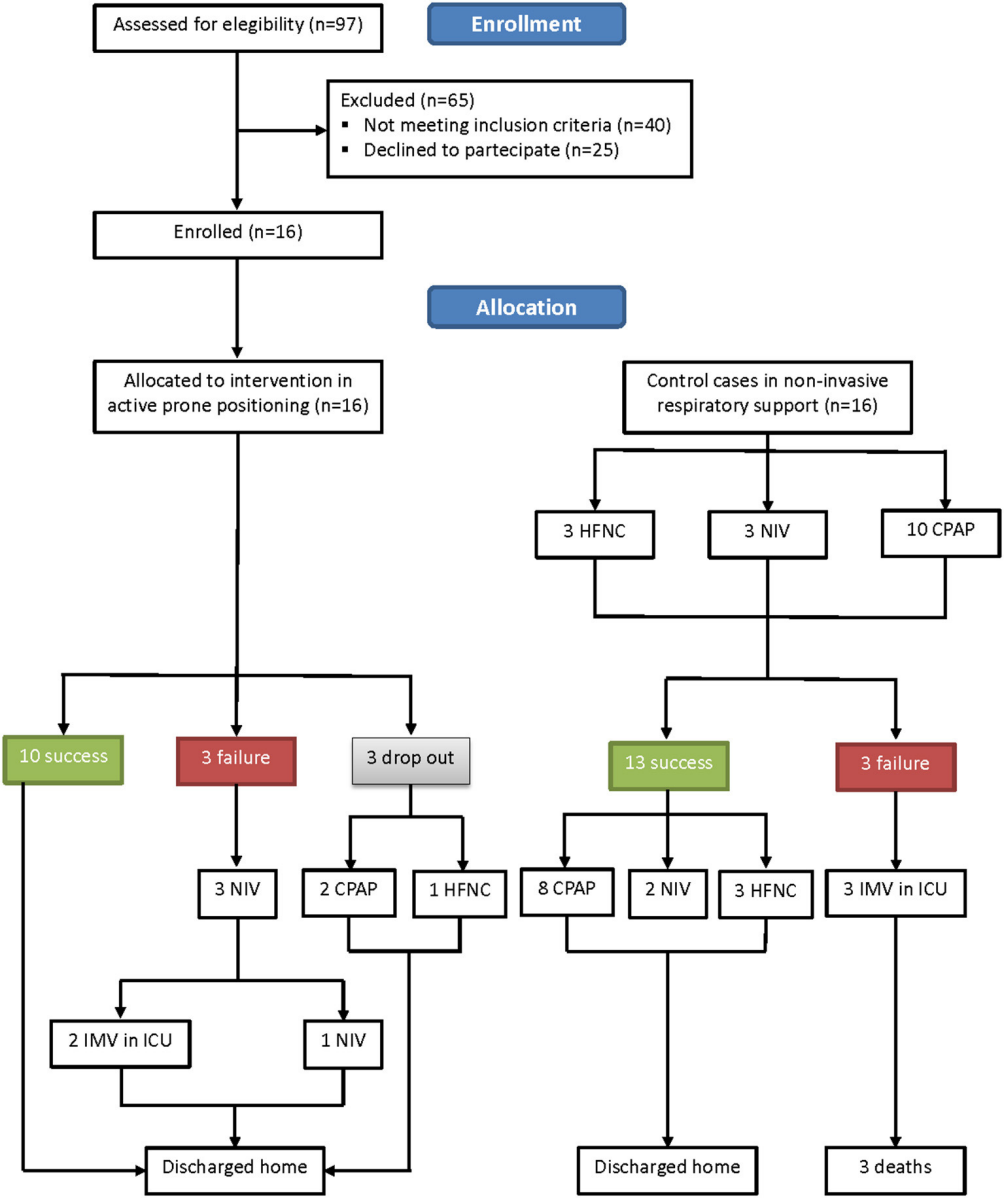
Study flow diagram.

Sixteen patients showed eligible criteria and agreed to be enrolled in the study. The other 16 patients were controls. The flow diagram of the study is shown in Figure 2. The demographic, anthropometric, and clinical characteristics of patients at admission are shown in Table 1. The baseline characteristics were similar between the two groups, with the exception of age, CRP, and PaCO_2_, with the controls being older, being more hypercapnic, and having a higher CRP.

**Table 1:** Demographic, physiological, and clinical characteristics of patients at RICU admission.

**Variables**	**Study group**	**Control group**	***p*-value**
Number	16	16	
Age, years	59 ± 11	70 ± 15	0.01
Males, *n* (%)	13 (81)	10 (62)	0.71
Smokers, *n* (%)	9 (56)	8 (50)	1
Presence of comorbidities, *n* (%)	13 (81.2)	13 (81.2)	1
Obesity, *n* (%)	5 (27.8)	4 (25)	1
Hypertension, *n* (%)	5 (31.2)	11 (68.8)	0.07
Diabetes, *n* (%)	3 (18.8)	4 (25)	1
Chronic cardiac failure, *n* (%)	0	3 (18.8)	0.22
Ischemic cardiac disease, *n* (%)	2 (12.5)	2 (12.5)	1
Chronic kidney disease, *n* (%)	5 (31.2)	6 (37.5)	1
Respiratory disease, *n* (%)	0	2 (12.5)	0.48
Autoimmune diseases, *n* (%)	2 (12.5)	0	0.48
SOFA*	2 [0.25]	2 [0]	0.61
PaO_2_	142 ± 62	127 ± 27	0.46
FiO_2_	57 ± 7	59 ± 13	0.31
PaO_2_/FiO_2_	226 ± 74	179 ± 18	0.11
PaCO_2_, mmHg	37 ± 4	43 ± 6	0.03
CPR, mg/L	78 ± 63	137 ± 80	0.03
WBC, 10^3^ μl	6.8 ± 2.4	8.9 ± 4.9	0.83
NLR	6.6 ± 3.8	8.8 ± 6.5	0.13
HR, bpm	78 ± 10	80 ± 12	0.38
RR, bpm	20 ± 4	20 ± 3	0.32
VR	1.6 ± 0.5	1.5 ± 0.8	0.46
Time from ED to RICU admission, days	11 ± 7	10 ± 10	0.47

Values are shown as mean ± SD, respectively, for normal distributed numeric variables or as median [IQR] for non-normal distributed numeric variables (*), and with % for categorical ones.

*SOFA, sequential organ failure assessment; ED, emergency department; CPR, C-reactive protein; WBC, white blood cell; NLR, neutrophil-to-lymphocyte ratio; HR, heart rate; RR, respiratory rate; VR, ventilatory ratio; LUS, lung ultrasound; RICU, respiratory intensive care unit*.

**Figure 3:**
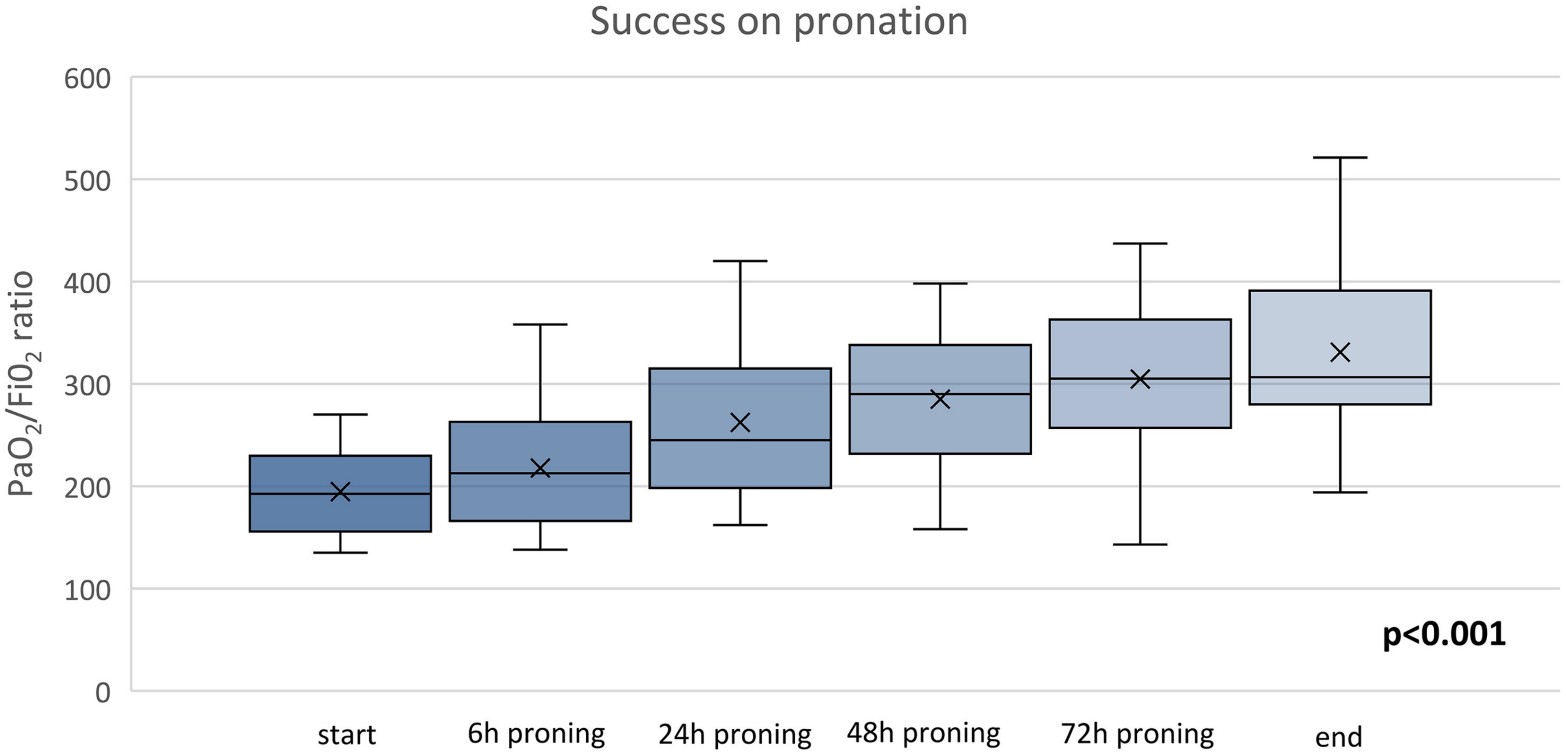
Time course of PaO_2_/FiO_2_ of PP group ANOVA, *p* = 0.001. The x refers to the average value and the continuous line refers to the median value.

Three out of 16 (18.7%) patients did not tolerate PP for chronic osteoarticular pain related to knee and spine degeneration; therefore, they received oxygen therapy in supine position. However, within 24 h from the discontinuation of PP, they all experienced worsening of their gas exchange. In more detail, one of them was supported *via* HFNC with FiO_2_ 0.75 and two were supported *via* helmet CPAP with FiO_2_ 0.70. None of them required intubation (Figure 2). Three more patients showed worsening in PaO_2_/FiO_2_ to <150 and underwent NIV; two of them finally needed endotracheal intubation. Ten out of 16 patients of the study group showed a significant improvement in oxygenation [PaO_2_/FiO_2_ from 194.6 (42.1) before to 304.7 (79.32) after 72 h of PP *via* ANCOVA test (*p* < 0.001)]. Figure 3 shows the individual and mean PaO_2_/FiO_2_ values during the trial in the successful patients of study group. The mean PaO_2_/FiO_2_ before discharge from the RICU was 330 ± 89.1.

As shown in Table 2, the baseline clinical characteristics of successful patients were not significantly different between groups with the exception of the PaCO_2_ being higher in controls. However, only two patients were affected by chronic respiratory failure in the control group before admission.

**Table 2:** Demographic, physiological, and clinical characteristics of successful patients at admission.

**Variables**	**Study group**	**Control group**	***p*-value**
	10	13	
Age, years ∧	60 ± 9	69 ± 17	0.07
Males, *n* (%)	8 (80)	10 (80)	1
Smokers, *n* (%)	5 (50)	6 (46)	1
Presence of comorbidities, *n* (%)	7 (70)	10 (77)	0.1
Obesity, *n* (%)	4 (40)	4 (30.8)	0.68
Hypertension, *n* (%)	4 (40)	8 (61.5)	0.41
Diabetes, *n* (%)	3 (30)	3 (23.1)	1
Chronic cardiac failure, *n* (%)	0	3 (23.1)	0.23
Ischemic cardiac disease, *n* (%)	1 (10)	2 (15.4)	1
Chronic kidney disease, *n* (%)	3 (30)	5 (38.5)	1
Respiratory disease, *n* (%)	0	2 (15.4)	0.48
Autoimmune diseases, *n* (%)	1(10)	0	0.43
SOFA*	2 [0.75]	2 [0]	0.12
PaO_2_	117 ± 25	117 ± 27	0.65
FiO_2_	60 ± 0	55.8 ± 10.6	0.42
PaO_2_/FiO_2_	195 ± 42	181 ± 19	0.17
PaCO_2_, mmHg	35 ± 3	42 ± 5	0.001
CPR, mg/L	84 ± 62	127 ± 70	0.09
WBC, 10^3^ μl	6.5 ± 2.6	8.7 ± 4.7	0.14
NLR	6.8 ± 3.4	8.7 ± 6.1	0.25
HR, bpm	76 ± 9	80 ± 12	0.29
RR, bpm	20 ± 4	21 ± 3	0.33
VR	1.5 ± 0.3	1.4 ± 0.4	0.43
Time from ED to RICU admission, days	13 ± 4	10 ± 10	0.40

Values are shown as mean ± SD, respectively, for normal distributed numeric variables or as median [IQR] for non-normal distributed numeric variables (*), and with % for categorical ones.

*SOFA, sequential organ failure assessment; ED, emergency department; CPR, C-reactive protein; WBC, white blood cell count; NLR, neutrophil-to-lymphocyte ratio; HR, heart rate; RR, respiratory rate; VR, ventilatory ratio; LUS, lung ultrasound; RICU, respiratory intensive care unit*.

The baseline VR was not different between the two groups. The LUS scores of the 10 successful patients changed from 13.0 [IQR 6.0–16.5] before, to 5.0 [IQR 2.5–12.0] at the end of PP (*p* = 0.0364), indicating a significant improvement in lung consolidations. All 10 patients were discharged home after a mean LoS of 21.0 (7.0) days (Figure 2). Patients undergoing active PP <12 days from hospital admission had a significantly shorter LoS as compared to the others [17.0 (5.0) vs. 25.0 (6.0) days, *p* = 0.04]. No patient who had undergone PP had died at 1- and 6-month follow-up.

**Figure 4:**
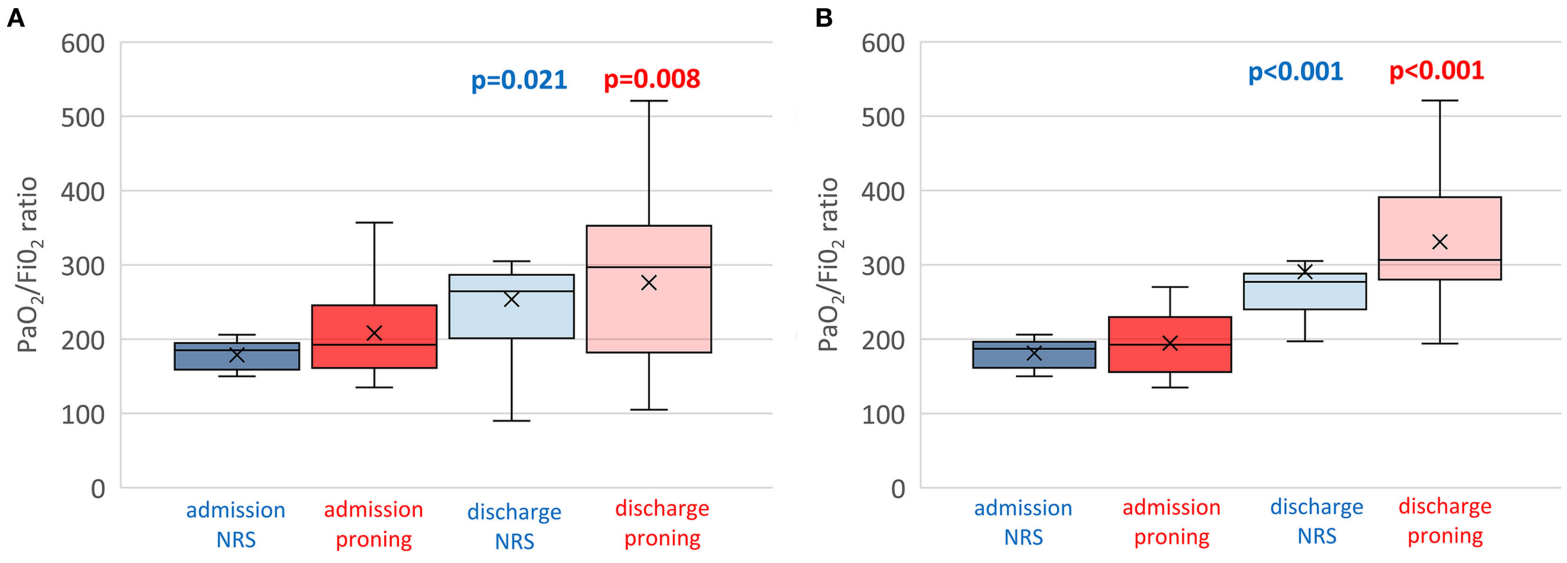
Individual (box plots) and time course of PaO_2_/FiO_2_ of both proning and control group on non-invasive respiratory support. **(A)** Total patients enrolled including dropouts and failures. **(B)** Only successful patients. Assessments at RICU admission and at RICU discharge. ANOVA, *p* = 0.001. The x refers to the average value and the continuous line refers to the median value.

As also shown in Figures 2, 4 out of 16 controls showed a worsening in PaO_2_/FiO_2_ to <150, requiring admission to ICU and endotracheal intubation. All three patients died in ICU within 1 month. The 13 successful controls showed a significant improvement in oxygenation from admission to discharge [PaO_2_/FiO_2_ from 181.2 (19.1) at the start to 290.5 (81.4) at discharge from RICU (*p* < 0.001)]. There was no significant difference in discharge to admission changes in PaO_2_/FiO_2_ between the successful patients of the study group or control group (*p* = 0.3156).

Figure 4A shows the individual and mean PaO_2_/FiO_2_ values at admission and discharge of all patients and controls while Figure 4B shows only successful patients including study group and controls.

Only one healthcare provider suffered from COVID-19 infection during the study period.

## Discussion

In this small single-center cohort study, we found that the use of PP in awake, spontaneously breathing patients with COVID-19-induced hypoxemic ARF with PaO_2_/FiO_2_ > 150 was feasible, tolerated for a prolonged time (three consecutive days), with similar results to patients undergoing NRS. The PP was associated with improved oxygenation and lung consolidations. Patients did not require ventilatory support in 62.5% of cases with good 1- and 6-month outcome.

Before the COVID-19 pandemic, there was limited published research on PP in non-intubated patients. The COVID-19 pandemic has led to a dramatic increase in patients requiring critical care, pushing clinicians to use new approaches to save resources for mechanical ventilation, including awake proning. A few physiological and clinical studies have evaluated the effects of PP in non-intubated patients with COVID-19 under NRS or spontaneously breathing (6–8, 8–10), and early helmet CPAP with moderate pressure and PP has been suggested (11).

This study enrolled unsupported breathing patients, and the active PP was tolerated all day long, with just short intervals for meals and personal care, for 3 consecutive days: to the best of our knowledge, this is the longest reported period: in literature, up to 8 daily hours of active PP are reported (9).

During the PP, the expansion of the anterior chest wall is limited, resulting in more homogeneous chest wall compliance and gravitational forces on lung parenchyma. This enables greater recruitment of the posterior zones, recruiting a larger proportion of alveoli clusters to participate in gas exchange. The diaphragm also greatly contributes to improve stress forces during PP, which may help to reduce lung injury during spontaneous breathing (17, 18). Indeed, the PP enhances the inferior movement of the diaphragm, which relieves compression on posterior lung zones that are usually atelectatic during supine positioning, thus improving their recruitment (19, 20).

The Ventilatory ratio (VR) is considered an index of respiratory effort, and it has been found to be independently associated with mortality in non-COVID-19 ARDS patients (21). In our patients, the mean VR was similar to survivors of non-COVID ARDS (13). Further studies will be required to confirm the VR ranges within COVID-19 patients.

Active PP may result in good physiological and clinical results, avoiding contacts and waste of human, economic, therapeutic resources and personal protective equipment (12, 14, 15). These patients were able to maintain active PP, and the only additional nursing workload was to move the EKG patches from the front to the back of the patients' chest.

The two groups of patients analyzed were similar but showed one significant difference in terms of age with the control group being older. Furthermore, the control group showed higher CRP and PaCO_2_ compared to the other. This, on one hand, would potentially expose the control group to a better response to NRS; on the other hand, both the SOFA score and the P/F ratio did not differ between the two groups, showing a substantial similar prediction of mortaility and grade of severity of hypoxic respiratory failure.

The setting of the study allows its repeatability in a clinical unit with patients under 24 h monitoring and adequate nurse support and supervision. Although promising, our case series should be interpreted with caution. In this selected group (PaO_2_/FiO_2_ > 150), three patients did not tolerate the PP, and two patients required intubation. The RICU is the right location for these unstable patients where adequately trained personnel may promptly recognize the need for treatment escalation and switch to NIV or IMV, respectively (22). Although improved oxygenation with the PP is important, hypoxemia has not been a reliable surrogate biomarker for mortality in clinical trials of ARDS (23).

This study has limitations. The sample size of patients was small (16 patients vs. 16 controls), and the data of controls were retrospectively collected. The performance of a chest CT scan in all admitted patients with hypoxic ARF would have added important information on the status of the lung parenchyma. However, due to the large number of patients to manage at that time, this was not promptly available for all admitted patients.

A control population of consecutively admitted patients only on low flow oxygen not undergoing NRS would have been more suitable for adequate comparison; however, at the time of the study, patients with PaO_2_/FiO_2_ > 150 used to be initiated to NRS.

The choice of SpO_2_/FiO_2_ would have been useful as a surrogate evaluation of time points in the initial 24 h; however, this parameter, although monitored, was not recorded.

On the other hand, our study highlights the feasibility, tolerability, and effectiveness of prolonged PP. Indeed, the shorter LoS of patients undergoing earlier PP suggests greater effectiveness in early application. Within the 6-month follow-up, no deaths occurred in the group that underwent PP. Furthermore, in consideration of the high shortage of ICU beds, the PP may represent a first-line treatment outside the ICU.

Spontaneously breathing patients with hypoxemic ARF may generate relatively large tidal volumes with potential self-inflicted lung injury (SILI) (24). Therefore, continuous monitoring of paradoxical breathing pattern and vital parameters should be warranted in these patients as they may deteriorate very fast. These concerns should be balanced with the lack of ICU bed availability and the risks of mechanical ventilation, including the need for prolonged sedation and the risk of ventilator-associated pneumonia.

## Conclusion

In conclusion, in the majority of the studied non-intubated spontaneously breathing series of patients with COVID-19-associated hypoxemic ARF with PaO_2_/FiO_2_ ratio >150 prolonged active PP was feasible and well-tolerated and associated to improvement in PaO_2_/FiO_2._ This approach may be confirmed by larger randomized controlled studies.

## Data Availability Statement

The raw data supporting the conclusions of this article will be made available by the authors, without undue reservation.

## Ethics Statement

The studies involving human participants were reviewed and approved by Bari Policlinico Ethic committee. Written informed consent was obtained from the individual(s) for the publication of any potentially identifiable images or data included in this article.

## Author Contributions

PP, VD, and GC made substantial contribution to the conception and design of the work. MD, EB, and AP: data acquisition. SB, NA, and GC: analysis. NA and OR: interpretation. PP, VD, MD, SB, EB, and AP helped in drafting the article. NA, GC, and OR revised it critically for important intellectual content. All authors gave the final approval of the version to be published and agreed for the accuracy or integrity of any part of the work.

## Conflict of Interest

The authors declare that the research was conducted in the absence of any commercial or financial relationships that could be construed as a potential conflict of interest.

## References

[1] Johns Hopkins University. Covid-19 Dashboard by the Centre for Systems Science and Engineering (CSSE) at Johns Hopkins University. Available online at: https://coronavirus.jhu.edu/map.html (accessed October 30, 2020).

[2] Franco C Facciolongo N Tonelli R Dongilli R Vianello A Pisani L. Feasibility and clinical impact of out-of-ICU non-invasive respiratory support in patients with COVID-19 related pneumonia. Eur Respir J. (2020) 3:2002130. 10.1183/13993003.02130-2020 PMC7397952 32747398

[3] Di Lecce V Carpagnano GE Pierucci P Quaranta VN Barratta F Zito A. Baseline characteristics and outcomes of COVID-19 patients admitted to a Respiratory Intensive Care Unit (RICU) in Southern Italy. Multidiscip Respir Med. (2020) 15:704. 10.4081/mrm.2020.704 33282282 PMC7662452

[4] Gattinoni L Taccone P Carlesso E Marini JJ. Prone position in acute respiratory distress syndrome. Rationale, indications, and limits. Am J Respir Crit Care Med. (2013) 188:1286–93. 10.1164/rccm.201308-1532CI 24134414

[5] Ding L Wang L Ma W He H, Efficacy and safety of early prone positioning combined with HFNC or NIV in moderate to severe ARDS: a multi-center prospective cohort study. Critical Care. (2020) 24:28. 10.1186/s13054-020-2738-5 32000806 PMC6993481

[6] Elharrar X Trigui Y Dols A-M Touchon F Martinez S Prud'homme E. Use of prone positioning in nonintubated patients with COVID-19 and hypoxemic acute respiratory failure. J Am Med Assoc. (2020) 323:2336–8. 10.1001/jama.2020.8255 32412581 PMC7229532

[7] Sartini C Tresoldi M Scarpellini P Tettamanti A Carcò F Landoni G. Respiratory parameters in patients with COVID-19 after using non-invasive ventilation in the prone position outside the intensive care unit. J Am Med Assoc. (2020) 323:2338–40. 10.1001/jama.2020.7861 32412606 PMC7229533

[8] Paul V Patel V Royse M Odish M Malhotra A Koenig S. Proning in non-intubated (PINI) in times of COVID-19: case series and a review. J Intensive Care Med. (2020) 35:818–24. 10.1177/0885066620934801 32633215 PMC7394050

[9] Coppo A Bellani G Winterton D DI Pierro M Soria A Faverio P. Feasibility and physiological effects of prone positioning in non-intubated patients with acute respiratory failure due to COVID-19 (PRON-COVID): a prospective cohort study. Lancet Respir Med. (2020) 8:765–74. 10.1016/S2213-2600(20)30268-X 32569585 PMC7304954

[10] Scaravilli V Grasselli G Castagna L Zanella A Isgro' S Lucchini A. Prone positioning improves oxygenation in spontaneously breathing non-intubated patients with hypoxemic acute respiratory failure: a retrospective study. J Crit Care. (2015) 30:1390–4. 10.1016/j.jcrc.2015.07.008 26271685

[11] Longhini F Bruni A Garofalo E Navalesi P Grasselli G Cosentini R. Helmet Continuous Positive Airway Pressure and prone positioning: a proposal for an early management of COVID-19 patients. Pulmonology. (2020) 26:186–91. 10.1016/j.pulmoe.2020.04.014 32386886 PMC7190517

[12] World Health Organization. Severe Acute Respiratory Infections Treatment Centre Practical Manual to Set Up and Manage a SARI Treatment Centre and a SARI Screening Facility in Health Care Facilities. (2020).

[13] Morales-Quinteros L Schultz MJ Bringué J Calfee CS Camprubí M Cremer OL. Estimated dead space fraction and the ventilatory ratio are associated with mortality in early ARDS. Ann Intensive Care. (2019) 9:128. 10.1186/s13613-019-0601-0 31754866 PMC6872683

[14] Ippolito M Vitale F Accurso G Ozzo P Gregoretti C Giarratano A. Medical masks and respirators for the protection of healthcare workers from COVID-19 and other viruses. Pulmonology. (2020) 26:204–12. 10.1016/j.pulmoe.2020.04.009 32362505 PMC7184017

[15] NIH. Covid-19 Treatment Guidelines. Available online at: https://www.covid19treatmentguidelines.nih.gov/whats-new/ (accessed May 30, 2021).

[16] RCore Team. R: A Language and Environment for Statistical Computing. Vienna: R Foundation for Statistical Computing (2020). Available online at: https://www.r-project.org/ (accessed June 19, 2021).

[17] Scholten EL Beitler JR Prisk GK Malhotra A. Treatment of ARDS with prone positioning. Chest. (2017) 151:215–24. 10.1016/j.chest.2016.06.032 27400909 PMC6026253

[18] Pappert D Rossaint R Slama K Grüning T Falkeet KJ. Influence of positioning on ventilation–perfusion relationships in severe adult respiratory distress syndrome. Chest. (1994) 106:1511–6. 10.1378/chest.106.5.1511 7956412

[19] Telias I Katira BH Brochard L. Is the prone position helpful during spontaneous breathing in patients with COVID-19? J Am Med Assoc. (2020) 323:2265–7. 10.1001/jama.2020.8539 32412579

[20] Slessarev M Cheng J Ondrejicka M Arntfield R. Patient self-proning with high-flow nasal cannula improves oxygenation in COVID-19 pneumonia. Can J Anaesth. (2020) 67:1288–90. 10.1007/s12630-020-01661-0 32319029 PMC7172385

[21] Sinha P Calfee CS Beitler JR Soni N Ho K Matthay MA. Physiologic analysis and clinical performance of the ventilatory ratio in acute respiratory distress syndrome. Am J Respir Crit Care Med. (2019) 199:333–41. 10.1164/rccm.201804-0692OC 30211618 PMC6363976

[22] Karim HMR Burns KEA Ciobanu LD El-Khatib M Nicolini A Vargas N. Noninvasive ventilation: education and training. A narrative analysis and an international consensus document. Adv Respir Med. (2019) 87:36–45. 10.5603/ARM.a2019.0006 30830962

[23] Acute Respiratory Distress Syndrome Network Brower RG Matthay MA Morris A Schoenfeld D Thompson BT. Ventilation with lower tidal volumes as compared with traditional tidal volumes for acute lung injury and the acute respiratory distress syndrome. N Engl J Med. (2000) 342:1301–8. 10.1056/NEJM200005043421801 10793162

[24] Jardin F Delorme G Hardy A Auvert B Beauchet A Bourdariaset JP. Reevaluation of hemodynamic consequences of positive pressure ventilation: emphasis on cyclic right ventricular after loading by mechanical lung inflation. Anesthesiology. (1990) 72:966. 10.1097/00000542-199006000-00003 2190501

